# News and Notes

**Published:** 1907-05

**Authors:** 


### NEWS AND NOTES.

Lord Lister celebrated his eightieth birthday on April 4th.

Mrs. Osler, the mother of Dr. William Osler, died in Toronto on March 18 at the age of 100 years and 3 months.

A Cancer Research Fund has recently been established in Paris by an endowment of $20,000 from Baron Rothschild.

Dr. H. R. Slack has recently added a six-room cottage annex to the LaGrange Sanatorium for a male and emergency ward.

The nurses of Georgia will introduce a bill when the next legislature convenes, providing for a "State Board for Trained Nurses."

A ne hospital is to be erected on the Mount of Olives by evangelical residents of Jerusalem. The cornerstone was laid on Easter day.

The British Parliament has rejected, by a vote of 750 to 118, a proposal to make the metric system standard in the United Kingdom.

Having sold his interest in the Atlanta Journal-Record of Medicine, Dr. M. B. Hutchins retires from the management after May 1, 1907.

The South Georgia Medical Association held its annual meeting at Cordele, April 5th, after which a banquet was given by the local society.

The fourth annual meeting of the Philippine Islands Medical Association was held in Manila, February 27th, and March 2nd, 1907, inclusive.

Dr. and Mrs. Lloyd Garee, of Sutton, W. Va., were burned to death April the 3rd, on their wedding night, in the Riverview hotel, which was destroyed by fire.

---

The United State Government recently paid $100,000 for the Louisiana quarantine stations, and Federal quarantine was established on the first of April.

---

The Bulletin of the Virginia State Board of Health shows that the monthly death rate of the whites was about 1.73 and the colored 3.19—nearly double that of the whites.

---

The sanitary department has been instructed by the board of health to remove all trash and other refuse, in addition to garbage and ashes, from the yards of Atlanta residences.

---

Professor Carl Hess, the famous ophthalmologist of Wurzburg, Bavaria, will be the guest of the section on ophthalmology of the American Medical Association at the Atlantic City session in June.

---

The Society of the County of Westchester has celebrated its one hundred and tenth anniversary by a dinner at the Hotel Astor. The society was organized in 1797 and is said to be the oldest organization of its kind in the United States.

---

The State of Delaware has recently passed a law imposing 20 years in the penitentiary and castration as the punishment for rape. It seems remarkable that a State north of the Mason and Dixon line should be the first to establish a law.

---

Dr. L. G. Hardman, of Commerce, Ga., has presented, through the Jackson Medical Society, the town of Jefferson a monument to Dr. Crawford W. Long, the discover of anaesthesia. The monument is to be erected on the site of the office where Dr. Long performed the first operation under anaesthesia.

Beginning with the session for 1909-10, the requirement for admission to Yale Medical School will be two years of university work, which must include courses in inorganic chemistry, physics and general biology. Arrangements will be made, whereby, through a proper choice of subjects, the students may secure both the B. A. and M. D. degrees in six years.

---

The United States Civil Service Commission announces an examination on June 13-14, 1907, to secure eligibles from which to make certification to fill at least five vacancies in the position of medical interne (male), at $600 per annum each, with maintenance, in the Government Hospital for the Insane, Washington, D. C., and vacancies as they may occur in any branch of the service requiring similar qualifications.

---

The American Institute of Social Service has received from Or| Sommerfield, a physician and scientist of Berlin, a valuable anti-tuberculosis exhibit for the department of industrial hygiene in its Museum of Security. There are 45 vials containing as many different kinds of dust, mineral, animal and vegetable, produced in our various industries. The same number of photographs show how these various dusts appear under the microsope. Extremely realistic models in wax, colored to life, represent human lungs as they are affected by occupational dusts, other models show normal lungs for comparison; while still others show the effects of industrial poisons on the system.

---

We note with pleasure the advances of the Cincinnati Sanatorium, as shown in their thirty-third annual report. This private hospital, for the care and treatment of persons afflicted with nervous and mental disorders, has just completed the south wing of the main building, which is a very desirable addition to the facilities of the institution. During the year there has been a daily average of ninety-three patients, with thirty-seven per cent of recoveries and a mortality rate of only 5.96 per cent.

